# A New Method of Extraction of Cataract

**Published:** 1872-12

**Authors:** 


*A New Method of Extraction of Cataract.* By R. LIEBRICH, Ophthalmic Surgeon and Lecturer on Ophthalmology to St. Thomas Hospital. Reprinted from St. Thomas Hospital Reports, Vol. I. Philadelphia: Claxton, Remsen & Haffelfinger, 1873.

This little pamphlet contains a description of what seems to be a very feasible and proper method to be pursued in the extraction of Cataract. The author discards iridectomy in his operations and makes his cut entirely in the cornea, with the exception of the puncture and contra-puncture, which are just within the sclerotic. The wound, which is preferable to make downward, is not so curved as it has been the custom to make it, and its centre is from 1½ to 2 millimetres within the margin of the cornea. But two instruments are required—a small knife and a cystotome, which has a common Daviel's spoon at the other end. The author has faithfully tried the utility of his operation in a number of instances, and has carefully observed the result before presenting his proposition to the public. It seems to be a valuable suggestion, which will in many cases remove the difficulties and dangers of failure which have in so many instances attended the operation for extraction of cataract.

